# Complete Cubonavicular Coalition Associated with Midfoot Osteoarthritis

**DOI:** 10.1155/2020/8850768

**Published:** 2020-07-30

**Authors:** Anne Kummer, Eric Dugert, Mouas Jammal

**Affiliations:** Hôpital Intercantonal de la Broye (HIB), Payerne, Switzerland

## Abstract

*Introduction*. Cubonavicular coalitions represent a relatively rare condition with less than forty cases described in the literature, the majority of which are fibrocartilaginous. To our knowledge, cubonavicular osseous coalition associated with osteoarthritis of the midfoot has never been described. *Case Presentation*. We present the case of a 26-year-old man with bilateral Achilles tendinosis, in whom radiological studies show an incidental finding of a complete osseous cubonavicular coalition, as well as a partial osseous cubo-third cuneiform coalition and a fibrous band between the first and second cuneiforms of the right foot, associated with arthritic changes of the tarsometatarsal joint. A nonosseous calcaneocubonavicular coalition was found on the left foot. These multiples coalitions were asymptomatic in this case. *Discussion*. Cubonavicular coalition, even asymptomatic, can cause midfoot osteoarthritis in young patients. We may therefore suspect that the immobility of the cubonavicular joint causes additional stress on the midfoot.

## 1. Introduction

Tarsal coalitions can be osseous (synostosis) or nonosseous (synchondrosis or syndesmosis) [1]. Coalitions are most often congenital and are believed to result from a failure of differentiation and segmentation, probably of autosomal dominant inheritance [2]. The true incidence of tarsal coalitions is not known because the number of asymptomatic coalitions has never been studied extensively [3], but is estimated to be less than 1% of the population [3, 4]. Coalitions are bilateral in more than 50% of cases [4], even 80% according to Leonard [2]. The most common types are talocalcaneal and calcaneonavicular coalitions [3], which represent more than 90% of all tarsal coalitions [4]. Multiple coalitions have also been described [5–7]. Cubonavicular coalition represents an infrequent type, with only 38 cases described in the literature (Tables 1 and 2), among which only 10 cases were osseous coalitions (Table 1).

We report a case of cubonavicular complete coalition, associated with other incomplete midtarsal coalitions, and with degenerative changes of the tarsometatarsal joint. This is the first case in the literature which associates a complete osseous cubonavicular coalition with tarsometatarsal arthritis.

## 2. Case Presentation

A 26-years-old man presented to the senior author for evaluation of bilateral heel pain, more severe on the right foot, for five months. He played hockey and tennis as leisure sports and worked as a dairy-cheese maker.

The physical examination demonstrated normal alignment of the lower extremities, straight hindfoot with painless limited motion of the subtalar joint bilaterally. Elective pain was present on both sides at the insertion of the Achilles tendon. The first tarsometatarsal (TMT1) joint was hypermobile and painless bilaterally.

**Figure 1:**
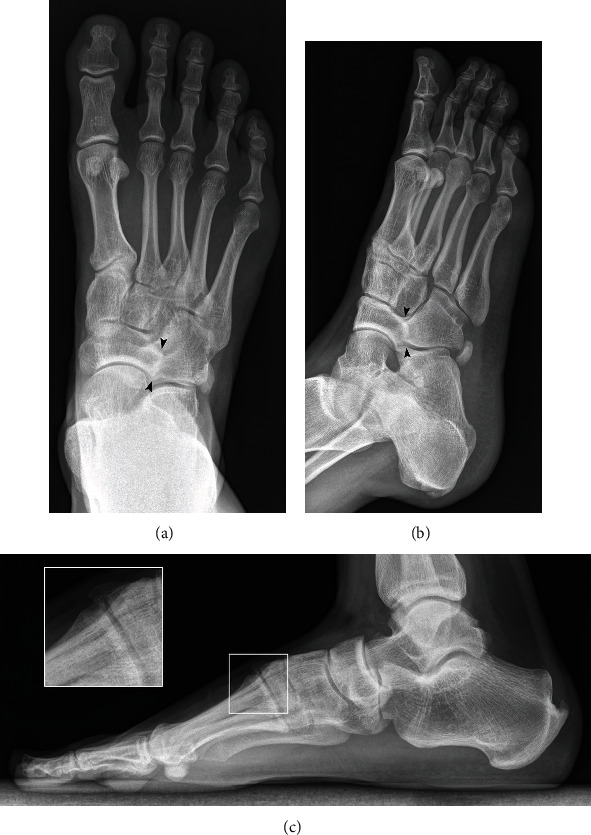
Frontal (a) and oblique (b) x-ray image of the right foot show an osseous cubonavicular coalition (arrowheads) with an absence of joint space. Lateral (c) x-ray image of the right foot shows degenerative changes with a spiky osteophytic deformation on the dorsal aspect of the first tarsometatarsal joint and a joint line irregularity (side shot).

**Figure 2:**
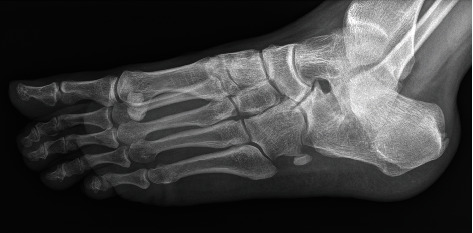
Oblique x-ray image of the left foot shows irregularity of the cubonavicular and calcaneonavicular articular surfaces with sclerotic changes.

Standard X-rays showed insertional Achilles calcific spurs, an incidental finding of osseous cubonavicular coalition on the right foot (Figures 1(a) and 1(b)), and irregularity of the cubonavicular and calcaneonavicular articular surfaces on the left foot (Figure 2). Arthritic changes were visible on the dorsal aspect of the first tarsometatarsal joint on both feet (Figure 1(c)). Medial foot arch was normal with a Djian-Annonier angle of 123° on both sides (angle defined between a line tangent to the inferior surface of the calcaneus and a line between the inferior point of talonavicular joint and the inferior point of the medial sesamoid, with normal value defined between 120 and 128° [8]).

**Figure 3:**
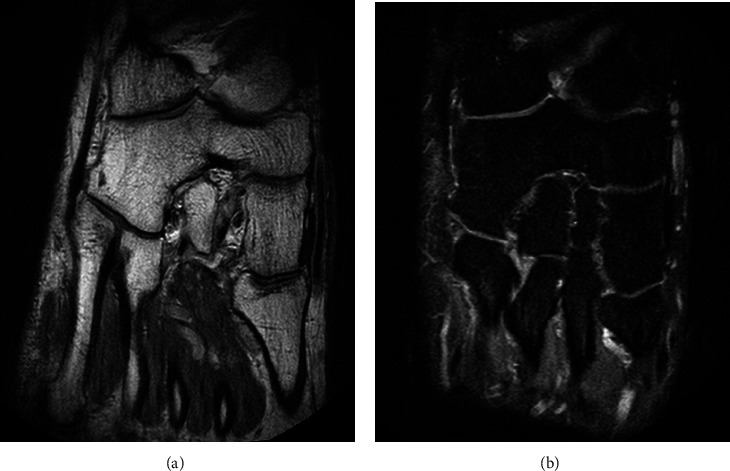
MRI of the right foot. Transversal T1-weighted (a) and PD-Fat Sat-weighted (b) images show a coalition between the cuboid and the navicular with a perfect continuity of the bone marrow and joint surfaces, without any bone oedema.

**Figure 4:**
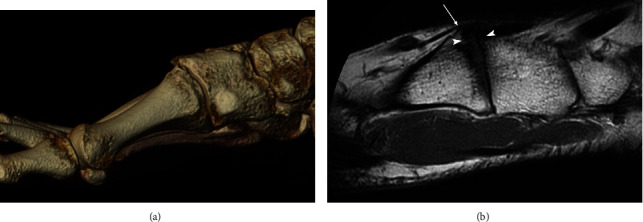
(a) Volume rendered CT image of the medial aspect of the right foot shows the same degenerative disorders as those shown in x-ray images, with a spiky osteophytic deformation on the dorsal aspect of the first tarsometatarsal joint. (b) Sagittal T1-weighted MRI image of the right foot shows a dorsal joint space narrowing (arrowheads), hypo T1 subchondral sclerosis and osteophyte formation (arrow).

**Figure 5:**
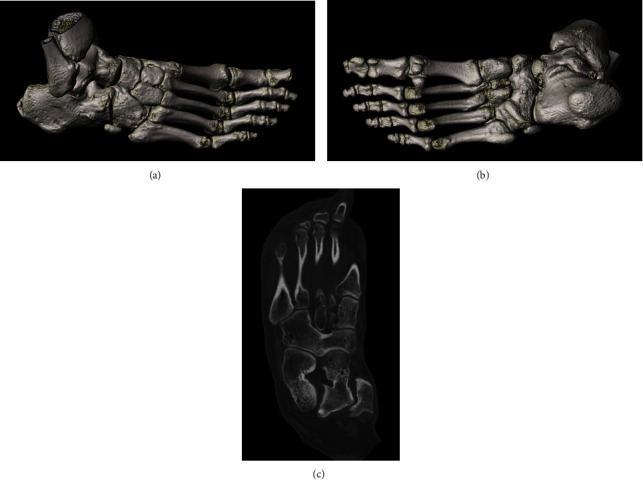
Osseous cubonavicular coalition of the right foot. Dorsolateral (a) and plantar (b) volume rendered CT images show the cuboidonavicular complex and its close relationship with the peripheral bones of the hindfoot and forefoot. Oblique multiplanar reconstruction CT image (c) shows as well as MRI the perfect continuity of the bone marrow and joint surfaces.

**Figure 6:**
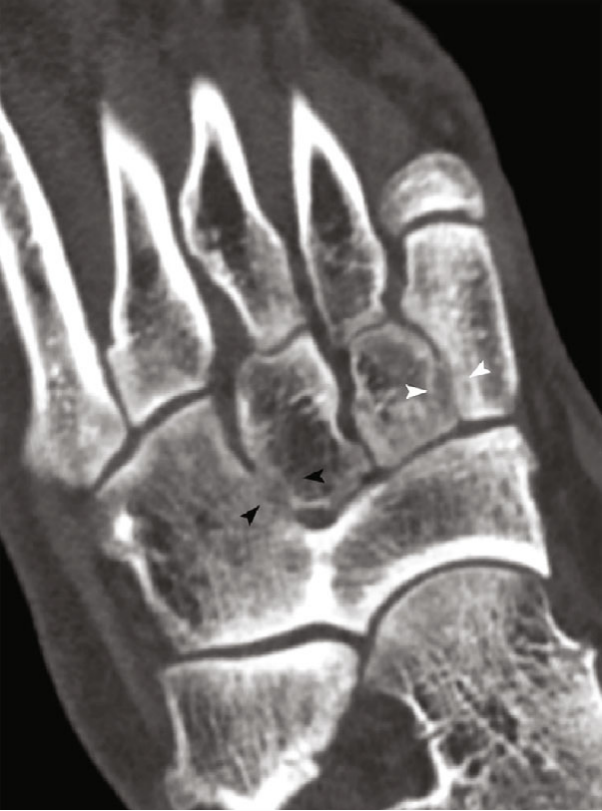
Oblique multiplanar reconstruction CT image of the right foot shows osseous cubonavicular coalition, associated with a partial osseous coalition between the cuboid and the third cuneiform with a focal lack of subchondral bone plate (black arrowheads) and bone irregularities between the first and second cuneiforms suggesting a nonosseous coalition (white arrowheads).

**Figure 7:**
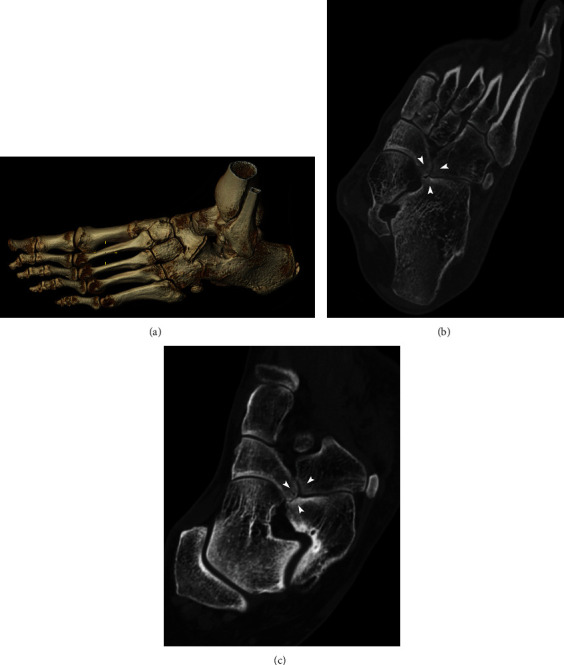
Dorsolateral volume rendered CT image of the left foot (a) shows hypertrophic arthritis changes between calcaneus and navicular due to nonosseous calcaneonavicular coalition. Oblique multiplanar reformatted CT images of the left foot (b-c) show a nonosseous calcaneonavicular coalition associated with a cuboidonavicular coalition (arrowheads), with degenerative changes between these tarsal bones.

MRI of the right foot showed insertional Achilles tendinosis and an osseous coalition between the navicular and the cuboid (Figure 3), as well as arthritic changes of the TMT1 (Figure 4(b)). Computed tomography of both feet was also performed and confirmed the osseous cubonavicular coalition on the right foot (Figure 5), associated with a partial osseous coalition between the cuboid and the third cuneiform and bone irregularities between the first and second cuneiforms suggesting a nonosseous coalition (Figure 6), as well as arthritis in the tarsometatarsal joint (Figure 4(a)). On the left foot, a nonosseous calcaneonavicular associated with a cubonavicular coalitions was diagnosed (Figure 7).

As the patient was not symptomatic of this midtarsal arthritis and of his rigid hindfoot, no surgical treatment was proposed. Insertional Achilles tendinosis was treated with physical therapy and foot orthoses with success.

## 3. Discussion

Classical tarsal coalitions can become symptomatic, usually during childhood or adolescence, causing stiffness and pain [3, 9]. Patients present typically with a history of ankle injury that is slow to resolve or diffuse pain exacerbated with activity, associated with difficulty to accommodate to uneven ground and repetitive ankle sprains [3, 10]. Clinical examination usually shows a diminished range of motion of the subtalar joint, rigid flat foot, tenderness over the site of the coalition, and possible peroneal spasm [3, 11]. Cubonavicular coalition can produce various clinical presentations, as summarized in Tables 1 and 2. Nonoperative treatment represents the first line therapy [1, 3, 11]. Surgical treatment is considered in case of failure of conservative measures, with coalition resection and interposition, or arthrodesis [1, 3, 10, 11]. Degenerative changes related to tarsal coalitions were described regarding arthritis involving the joint affected by the coalition, especially for talocalcaneal coalitions [12, 13]. In the case of symptomatic calcaneonavicular or talocalcaneal coalition with severe degenerative changes of these joints, triple arthrodesis is recommended [1, 3, 11, 14].

Cohen et al. studied an adult population with incomplete calcaneonavicular coalitions and reported approximately 75% of degenerative changes involving adjacent joints (naviculocuneiform, subtalar and talonavicular joints) on preoperative x-rays [15]. Regarding cubonavicular coalitions, only two authors, to our knowledge, mentioned degenerative changes of adjacent joints, both associated with a nonosseous coalition: talonavicular arthritis was reported by Ehredt et al. [16], and arthritis of the tarsometatarsal joint was mentioned by Sarage et al. [17]. However, in this latter case, the authors report neither x-rays nor other details regarding these arthritic lesions. Among the few described cases of osseous coalition (Table 1), none was associated with arthritic changes. We described therefore the first case of a cubonavicular osseous coalition associated with other incomplete coalitions and midfoot arthritis.

Considering the relationship between the subtalar and the midtarsal joints during the stance phase, the axis of the talonavicular and the calcaneocuboid joints become parallel with pronation [9, 18]; thereby, the cuboid and navicular are independent from one another [18]. According to Cavallaro and Hadden [18], restriction of the normal movement between the cuboid and navicular could have an irritative effect by interfering with the physiological mobility of the talonavicular, calcaneocuboid, and subtalar joints and would result in stabilization of the subtalar joint and overloading of midtarsal joints. We can postulate that, if the subtalar become more rigid because of a cubonavicular coalition, accommodation on the uneven floor is less effective and that mechanical stress could be transmitted to tarsometatarsal joint and causing osteoarthritis.

Pain associated with a tarsal coalition is suspected to be related to an incomplete coalition, where the union is particularly prone to motion and strain [15, 19]. However, a complete osseous coalition can also be symptomatic [20]. In our case, the patient was asymptomatic, despite early degenerative changes of the tarsometatarsal joint, and the cubonavicular coalition was an incidental finding. The review of the literature shows three cases of asymptomatic cubonavicular coalitions, found out by radiological assessment following other injuries: violence to both feet in one case [21], lateral ankle sprain in another [22], and tibial anterior tendon rupture for the third [23]. A fourth case of incidental finding was described by Chu [24], after a pilon fracture, but it is unclear whether or not the patient was symptomatic from the coalition. Interestingly, these three asymptomatic patients [21–23] were in their forties (mean and median 45 years old), while symptomatic patients [16–20, 25–35] were younger (mean age 23 years old, median 17 years old). The analysis of the cases of the literature for cubonavicular coalition revealed that symptoms were more frequent for nonosseous (14/16, 88%) than osseous (7/9 cases, 78%) union but the small number of cases does not allow any assertion.

This case of a complete osseous cubonavicular coalition with early osteoarthritic changes shows that clinical and radiological presentation of such coalition could be very different among patients. Our patient was asymptomatic, despite multiple tarsal coalitions and degenerative signs of the tarsometatarsal joint, while other patients report daily disabling pain, whether the coalition is bony or fibrous. In any case, surgical treatment should only be proposed if the coalition is symptomatic and after failure of conservative treatment.

## Figures and Tables

**Table 1:** Review of the published cases of osseous cubonavicular coalition.

Authors	Year	No. of cases	Sex	Age	Side	Nature of coalition	Symptoms	Physical examination
Waugh [19]	1957	1	M	15	L+R	O (L), NO (R)	Left foot pain with activity	Left peroneal spasm
Del Sel [21]	1959	1	M	43	L+R	O (L), NO (R)	None	Cavus deformity, slight inversion/eversion
Cavallaro [18]	1978	1	F	12	L+R	O (L+R)	Bilateral ankle pain with activity	Pain and stiffness of subtalar joint, painful sinus tarsi
O'Neill [36]	1989	1	F	15	R	O	n.a.	Limitation of inversion/eversion
Williamson [20]	1992	1	M	14	L+R	O (L+R)	Bilateral foot pain with activity	Bilateral hindfoot valgus, decreased subtalar motion, peroneal spasm
Talkhani [25]	1999	1	M	42	L	O	Midfoot pain with walking and morning stiffness	Diminished midfoot movements
Piqueres [26]	2002	1	M	14	L+R	O (L), NO (R)	Left midtarsal pain with weight bearing	Restriction of plantar flexion and eversion, valgus rearfoot, flat left foot
Johnson [27]	2005	1	M	15	L	O	Midfoot and ankle pain with activity	Decreased subtalar and transverse tarsal motion, fixed pes planus
Prado [28]	2010	1	F	9	R	O	Foot pain with exercises	Limitation of mobility of midtarsal joint
García-Mata [22]	2011	1	M	45	R	O	None	Pain over cuboid

M: male; F: female; L: left; R: right; O: osseous; NO: nonosseous; n.a.: nonavailable.

**Table 2:** Review of the published cases of nonosseous or undefined cubonavicular coalition.

Authors	Year	No. of cases	Sex	Age	Side	Nature of coalition	Symptoms	Physical examination
Cowell [37]	1982	1	n.a.	n.a.	L	NO	Pain with sport activities	Limited motion without details
Feliu [29]	1991	1	M	24	L	NO	Spontaneous pain dorsum of the foot	Normal
Palladino [30]	1991	1	M	13	L+R	NO (L+R)	Rearfoot pain with activity (right more than left)	Bilateral peroneal spasm, rigid subtalar joint, pes planovalgus
Newman [38]	2000	1	F	10	R	NO	n.a.	n.a.
Hounshell [31]	2011	1	F	37	L	NO	Persistent foot pain 8 months after a sprain	Decreased subtalar motion, painful sinus tarsi
Sarage [17]	2012	4	F	15	L	NO	Foot pain for 4 months with activity	Cuboid and navicular pain, decreased subtalar motion
			M	16	L+R	NO (L+R)	Bilateral dorsolateral midfoot pain	n.a.
			M	35	L	NO	Dorsolateral midfoot pain for 1 year	n.a.
			F	18	R	NO	Foot and ankle pain for 4 years with activity	Painful sinus tarsi, normal range of motion
De Keyzer [32]	2013	1	F	40	R	NO	Mechanical pain for some duration	Decreased subtalar motion, pes planovalgus
Lawrence [33]	2014	2	M	40	R	NO	Chronic midfoot pain	n.a.
			M	44	R	NO	Vague ankle pain	n.a.
Awan [34]	2015	1	M	17	R	NO	Foot pain for 6 months with activity	Tenderness over tarsonavicular region
Kamiya [35]	2015	1	F	14	R	NO	Midfoot pain exacerbated with activity	Normal
Chu [24]	2017	1	F	34	R	NO	Not clear (x-rays for distal tibia fracture)	Symptoms of post-traumatic arthritis (localization n.a.)
Berger-Groch [23]	2018	1	M	47	L	NO	None	Pes valgus and abductus
Ehredt [16]	2020	1	M	34	L	NO	Dorsolateral midfoot pain for 2 years with activity	Pes planovalgus, decreased subtalar motion
Harris [39]	1965	1	n.a.	n.a.	n.a.	n.a.	n.a.	n.a.
Rankin [40]	1974	1	n.a.	n.a.	n.a.	n.a.	n.a.	n.a.
Stormont [4]	1983	1	M	26	L	n.a.†	n.a.	n.a.
Sarrafian [41]	2011‡	8	n.a.	n.a.	n.a.	n.a.	n.a.	n.a.

^†^naviculo-cubo-third cuneiform (x-rays n.a.). ^‡^4 cases reported by Gruber in 1871, 3 cases reported by Pfitzner in 1896, 1 case reported by Cruveilhier (1829-1835). M: male; F: female; L: left; R: right; NO: nonosseous; n.a.: nonavailable.
